# Preparation and Modification of Porous Polyetheretherketone (PEEK) Cage Material Based on Fused Deposition Modeling (FDM)

**DOI:** 10.3390/polym14245403

**Published:** 2022-12-09

**Authors:** Hui Zhang, Mingde Duan, Shikun Qin, Zhuangya Zhang

**Affiliations:** School of Mechatronics Engineering, Henan University of Science and Technology, Luoyang 471003, China

**Keywords:** FDM, PEEK, bearing cage, heat treatment, self-lubrication

## Abstract

To address the problems of the difficult processing and internal microstructure disorder of porous bearing cages, Polyetheretherketone (PEEK) porous self-lubricating bearing cage material was prepared based on a fused deposition molding (FDM) process, and the porous samples were heat-treated on this basis, the research was carried out around the synergistic design of the material preparation, microstructure, and tribological properties. The results show that the pore size of the PEEK porous material prepared by the FDM process meets the requirements of the porous bearing cage; the samples with higher porosity also have higher oil content, and all the samples show high oil retention. Under dry friction conditions, the higher the porosity of the porous material, the larger the friction coefficient, and the friction coefficients of each sample after heat treatment show the same pattern; under starved lubrication conditions, the friction coefficient of the porous PEEK material decreased significantly compared to the compact PEEK material, showing a better self-lubrication effect, and the porous samples reached the best self-lubrication effect after heat treatment. The optimal process parameters were 60% mass fraction of NaCl, 40% mass fraction of PEEK, and the applied heat treatment process.

## 1. Introduction

As key components in spacecraft, the lubrication performance of bearings and bearing units directly determines the precision, life, and reliability of the spacecraft [1]. Study showed that there were dozens of satellite failure cases caused by the lubrication failure of bearing components [2]. Most of the bearing lubrication failure cases were triggered by cage-guiding edge wear, and the running instability of the cage can easily cause serious wear of bearing bodies, accompanied by whirling and squealing [3]. Especially for special applications such as aerospace bearings, where the working conditions are complex and the lubricant cannot be replenished, conventional lubrication technology has failed to meet these needs.

Scholars generally believe that the bearing cages prepared based on porous self-lubricating materials can bring a chance to solve the above problems. Immersing the lubricant or grease into the porous cage pores, when bearings are running, the self-lubricating effect of the friction pair could be realized under the synergistic effect of centrifugal force, temperature rise, and other factors to achieve friction reduction and an anti-wear purpose [4,5,6]. Common self-lubricating bearing cage materials are cotton-phenolic, nylon (PA), polyimide (PI), polyether ether ketone (PEEK), etc. [7].

The porous structure of cotton fiber makes the cotton-phenolic material have certain oil storage, oil supply, and oil return functions, so that the porous cotton-phenolic material can be used as a high-speed bearing cage material, such as the type 125 gyro bearing cage used in the British Ferranti defense system. However, Bertrand [8] showed that the internal pores of this type of material are rich in hydrophilic functional groups (such as hydroxyl) and have strong hygroscopicity, resulting in a significant decrease in the oil content of the material. Therefore, hygroscopicity becomes one of the key factors affecting the service life and working precision of cotton-phenolic bearings. Porous nylon cage material has the advantages of adequate raw materials, low cost, and easy processing, and has been applied to the U.S. Titan-2 gyro bearing as early as 1963. However, the size stability, impact resistance and wear resistance of nylon materials are poor. These defects have limited its wide application in the aerospace field. Additionally, Bertrand [9] showed that, although the hygroscopicity of nylon is a little weaker than cotton-phenolic material, it still affects the working precision of bearings. PI has the advantages of high- and low-temperature resistance, irradiation resistance, a low expansion coefficient, etc., and has a wide range of application prospects in high-end technology fields such as aviation and aerospace [10,11,12]. However, PI molecules contain a large number of imide rings and benzene ring structures on their main chains, and there are large interaction forces between molecular main chains, resulting in poor melt flow behavior and difficulty in processing. Polyether ether ketone (PEEK) is a special engineering plastic that combines high strength, high toughness, high heat resistance, and other excellent overall performance. In addition to the above advantages, PEEK also has outstanding tribological properties [13,14,15,16]. It has excellent sliding wear resistance and fretting wear resistance and maintains high wear resistance and a low friction coefficient at 250 °C. In addition, PEEK has excellent processing properties, is easy to extrude or inject when molding with high molding efficiency, and has a wide range of applications in petrochemical, aerospace, and other fields.

Currently, the preparation of porous self-lubricating bearing cage materials is mainly performed by a cold-pressing sintering process and a mold-leaching process [17,18]. The cold-press sintering process is to sinter polymer particles by mechanical compaction at a certain temperature, and the polymer particles are bonded together by melting the surface so that the gaps between the particles form pore channels. Qiu [19] prepared porous PI materials using a cold-press sintering process and researched the effects of different porosities and lubricant types on the tribological properties of porous PI materials. The results showed that the porous PI material with 26% porosity had an “ink-bottle” pore structure, and the friction coefficient of the porous PI material with lubricant P200 decreased with increasing speed, while the friction coefficient of the porous PI material with lubricants M1001 and M1 increased with increasing speed. Jia [20] used polystyrene (PS) microspheres as a porogenic agent and mesoporous silica nanotubes (MSNT) as filler to modify porous (PI), and prepared porous PI/MSNT composite films by the cold-pressing sintering process, and then researched the effects of MSNT addition on the thermal stability, oil storage, mechanical properties, and tribological properties of porous PI films. It was shown that the addition of MSNT improved the thermal stability and mechanical properties of the porous PI matrix compared to the single-component PI oil-containing films. The mold-leaching process is a method to construct porous materials by the in situ removal of the porogenic agent after mixing polymer particles with filler and cold-pressing sintering. Wang [21] prepared porous PEEK sweat-based self-lubricating materials by the mold-leaching process and investigated the effects of the molding pressure, porogenic agent content, and type of lubricating grease on the frictional wear properties of PEEK porous self-lubricating materials, respectively. The study showed that the wear resistance of PEEK was increased by 1245 times compared to compact PEEK material under dry friction, the porous structure could store lubricant and form a stable and continuous oil film on the counterpart surface by sweating during the friction process, thus significantly reducing the friction coefficient and wear rate of the composite. Yan [22] prepared porous PI materials based on the mold-leaching process and investigated the effect of porogenic agent content on the mechanical and tribological properties of porous PI materials. The results showed that the porosity of the porous PI materials increased with the increase in the porogenic agent content, and the effect of the porogenic agent content on the friction coefficient was more obvious under sufficient lubrication conditions. Although the above preparation process has the advantages of simple operation and low cost, it is still difficult to produce porous materials with orderly pore arrangement, controllable porosity, and a variety of pore shapes.

The flourishing development of additive manufacturing technology represented by fused deposition molding (FDM) provides an effective solution for the preparation of porous materials with complex structures [23]. The FDM process can prepare porous cage materials with excellent properties in an efficient and green way based on the principle of layered manufacturing and layer-upon-layer [23,24,25,26]. Furthermore, great progress has been made in research related to improving the properties of polymers through fiber reinforcement and other means, but less research has been conducted to improve the mechanical properties of polymers through heat treatment processes. Yang [27] prepared PEEK samples based on a temperature-controlled 3D printing system and performed crystallinity and mechanical property tests. The relationship between various heat treatment conditions in the FDM process and the crystallinity and mechanical properties of the compact PEEK samples was investigated. The results showed that the crystallinity of the PEEK material increased from 17% to 31% as the ambient temperature increased from 25 °C to 200 °C, and the mechanical properties were closely related to the ambient temperature. Wang [28] prepared PEEK samples based on the FDM process and investigated the effect of heat treatment process parameters on the mechanical properties of PEEK samples. The results showed that the implementation of the heat treatment process could effectively improve the tensile strength of the samples, and this effect was through improving the crystallinity of the PEEK material, independent of the porosity change. All of the above studies point out that reasonable heat treatment process parameters can effectively improve the degree of crystallinity of the PEEK material, which is an important factor affecting its tribological properties. Therefore, research on improving the tribological properties of the polymer-bearing cage materials by the heat treatment process is worth looking forward to.

In this paper, the fused deposition-water washing method was proposed to prepare PEEK-based porous self-lubricating bearing cage material. Based on this process, the porous self-lubricating bearing cage material was prepared and subjected to heat treatment. The influence of the microstructure of porous self-lubricating materials on the tribological properties under different preparation parameters was studied, which provides a basis for the integrated design and manufacture of the material-structure performance of a porous self-lubricating bearing cage.

## 2. Materials and Methods

### 2.1. Composite Filament and Sample Preparation

(1) Composite filament preparation

Using PEEK as the base material and NaCl as the porogenic agent, the PEEK-based composite filament was prepared through the processes of powder mixing and filament extrusion.

The PEEK powder was produced by Jilin Joinature Polymer Co., Ltd. with a particle size of 200 mesh, a density of 1.3 g/cm^3^, and a melting point of 334 °C. The NaCl powder was produced by Shanghai Macklin Biochemical Co., Ltd., with a density of 2.1 g/cm^3^ and a melting point of 804 °C. The NaCl was passed through a 325-mesh sieve.

NaCl and PEEK powder were mixed with the mass ratio according to Table 1, and the powder was dried in an electric tachometer indicator thermostatic drying oven (202-0, Longkou Boiler Manufacturing Factory, LongKou, China) at 120 °C for 12 h. The powder was mixed mechanically at 50 r/min for 12 h, and the mixed powder was dried twice according to the above drying process parameters. After filament traction, cooling, measurement, and winding, the composite filament with a diameter of (1.75 ± 0.05) mm was prepared. To summarize the experience of the previous extensive experimental research, for the different formulations of filaments, the temperature and screw speed settings of the seven heating zones in the twin-screw extruder (YTG-20, Guangzhou Yongtuo Plastic Machinery Factory) are shown in Table 2.

To avoid NaCl dehydration or agglomeration, the filaments were placed in an electric tachometer indicator thermostatic drying oven at 60 °C for printing.

(2) Sample printing

**Figure 1:**
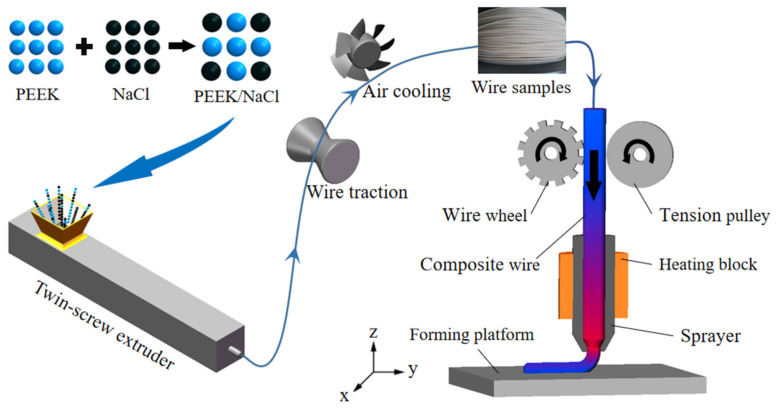
Schematic illustration of the FDM process.

Using an FDM rapid prototyping machine (ENGINEER Q300, Shaanxi JuGao-AM Co., Ltd., Weinan, China), choose the nozzle with a 0.8 mm diameter. Set the FDM process parameters as follows: nozzle temperature of 420 °C, printing speed of 40 mm/s, print layer thickness of 0.4 mm, and fill rate of 100%. The FDM process is shown in Figure 1.

(3) Heat treatment of samples

Selecting samples for tribological performance testing to be heat treated: the printed samples were placed in an oven with a heating rate of 8 °C/min and heat preservation for 2 h at 300 °C. Heat-treated composite samples were obtained.

### 2.2. Structural Characterization and Performance Testing

(1) Structural characterization

The pore size and porosity of porous materials are the key parameters to describe their microstructure. To explore the internal microstructure of porous samples, a laser scanning confocal microscope (LSCM, Zeiss-LSM800, Oberkochen, Germany) was used to observe the microscopic morphology of porous PEEK samples; the effect of NaCl removal inside the sample was tested using field emission scanning electron microscopy (EDS, JSM-IT800 type from Electronics Corporation, Tokyo, Japan); a mercury porosimeter (Mike, AutoPore IV 9500, York, PA, USA) was used to determine the pore size distribution and porosity of the porous PEEK samples.

(2) Performance testing

In order to research the macroscopic properties of the porous samples, a high-temperature friction and wear testing machine (Lanzhou Zhongke Kaihua Technology Development Co., Ltd., Model HT-1000, Lanzhou in China) was used to test the friction and wear properties of the material under dry friction and starved lubrication. Set the test conditions as follows: the diameter of the grinding ball is 5 mm, the steel ball material is 9Cr18, the applied load is 5 N, the friction radius is 5 mm, the speed is set to 392 r/min, and the test time is 30 min. Adopt a bearing cage vacuum oil immersion machine (Luoyang Bearing Research Institute Co., Ltd., ZKXY-400 type, Luoyang in China) to press the lubricant into the samples, and the oil retention of the sample was tested by centrifuge (Hunan Xiang Yi Laboratory Instrument Development Co., Ltd., TG16-WS, Changsha in China). The oil content and oil retention were calculated as follows.

(1) $$\mathrm{Oil}\;\mathrm{content}=\frac{m_{0}-m_{0}^{\prime }}{m_{0}^{\prime }}\times 100\%$$

(2) $$\mathrm{Oil}\;\mathrm{retension}=\frac{\mathrm{Oil}\;\mathrm{content}\;\mathrm{of}\;\mathrm{the}\;\mathrm{sample}\;\mathrm{after}\;T\;\mathrm{minutes}\;\mathrm{of}\;\mathrm{oil}\;\mathrm{throwing}}{\mathrm{Original}\;\mathrm{oil}\;\mathrm{content}\;\mathrm{of}\;\mathrm{the}\;\mathrm{sample}}\times 100\%$$

In the equations, $m_{0}^{\prime }$ is the weight of compact porous PEEK; $m_{0}$ is the weight of porous PEEK impregnated with lubricants.

The samples were cleaned, weighed, and immersed in lubricating oil (70 °C, 48 h) under vacuum (1.0 × 10^−3^ Pa) so that the internal pores were filled with lubricating oil. The samples were taken out and drained at normal temperature (25 °C) for 48 h and weighed, and the masses of the samples before and after oil immersion were obtained. The centrifuge was used to turn the oil-containing samples at normal temperature (25 °C) for 2 h (coaxial rotation, speed 8000 r/min). The mass of the samples was measured before oil immersion and after oil splashing, and the oil retention of the samples was calculated by Equation (2).

## 3. Results

### 3.1. Surface Morphology Characterization of Composite Filaments

**Figure 2:**
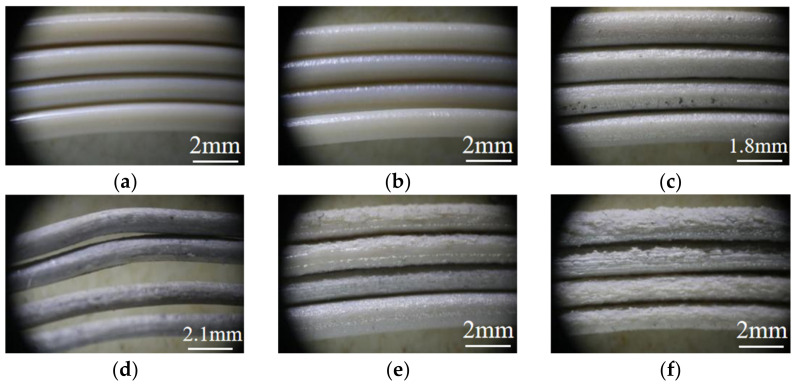
The morphology of the filament corresponding to each sample. The filament corresponds to the following samples: (**a**) Sample 1#; (**b**) Sample 2#; (**c**) Sample 3#; (**d**) Sample 4#; (**e**) Sample 5#; (**f**) Sample 6#.

With the increase in NaCl content, especially after the mass fraction of NaCl reached 70−80%, it was difficult to form the filaments normally with the process parameters corresponding to the low NaCl content. After adjusting the heating temperature and reducing the screw rotation speed, the surface morphology of each sample corresponding to the filaments is shown in Figure 2.

It can be seen that as the porogenic agent content increases, the surface roughness of the filament material also gradually increases, and the control of the filament diameter would be affected. This is because the lower the NaCl content, the better the dispersion effect in the matrix material, and the particles can be uniformly distributed in the matrix material. Therefore, the lower the NaCl content, the better the filament surface finish and the more flexible it was, while as the NaCl content increased, the continuity of NaCl in the matrix material was stronger, and the consequent agglomeration phenomenon was more obvious, forming a higher roughness and less flexible filament.

Elongation at break refers to the ratio of the elongation of a filament to its initial length after it has been tensioned until it breaks. It can be used to characterize the flexibility of PEEK filaments in this study. The greater the elongation at break, the better the flexibility of the filament, and the worse the opposite. Five sets of tests were performed for each ratio, and their elongations at break were distributed and averaged as shown in Table 3.

It is easy to conclude from the data in the table that sample 1# corresponds to the best flexibility of the filament. As the mass fraction of NaCl increases, the flexibility of the filament material appears to decrease significantly, while sample 6# corresponds to the lowest flexibility of the filament material.

### 3.2. Microstructural Characterization of Porous Samples

To reveal the mechanism of oil content and friction reduction in PEEK-based porous materials, laser confocal tests were conducted on molded porous structural parts to characterize their surface morphology and pore characteristics.

**Figure 3:**
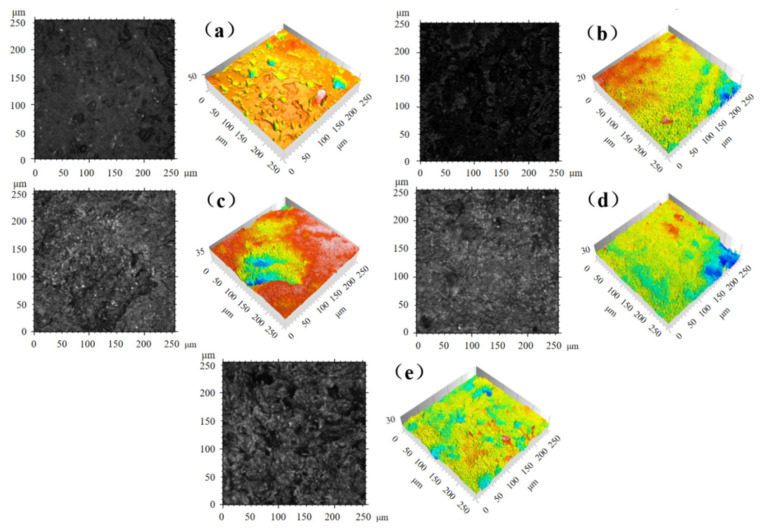
Two-dimensional and three-dimensional morphology of porous samples: (**a**) Sample 2#; (**b**) Sample 3#; (**c**) Sample 4#; (**d**) Sample 5#; (**e**) Sample 6#.

The microscopic pore distribution and pore size inside the porous material are the key factors affecting its macroscopic properties. It can be seen from Figure 3 that the pores left by the in situ elimination of NaCl were distributed inside the matrix material uniformly. The pore distribution and pore size of the samples were more reasonable, and the skeleton was more uniform as the percentage of NaCl increased. This is due to the excellent pore-forming ability of NaCl and the uniform size of the sieved NaCl particles, which could be used as a porogenic agent to prepare composites with abundant pores and controllable pore size [5]. After EDS analysis, Table 4 shows the results of the elemental percentage content of porous samples after NaCl removal. It can be seen that under the laboratory setting conditions, NaCl can be basically completely removed, which ensures the porogenic effect of porous samples to a certain extent, and also effectively avoids the adverse effects on the rolling body, inner and outer rings, and other parts.

An appropriate pore size facilitates the migration and recovery of lubricant in the cage pore during bearing operation. The results of numerous experimental studies have shown that the pore size of porous materials used for bearing cages should be less than 10 μm [29].

**Figure 4:**
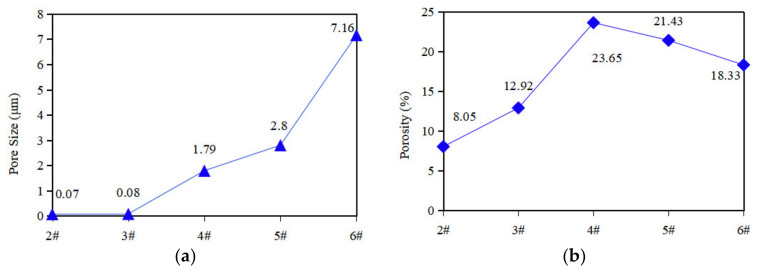
Pore size and porosity of porous samples. (**a**) Pore size of 2#~6# samples; (**b**) Porosity of 2#~6# samples.

To further explore the porosity effect of the porous samples, the results of the porosity and pore size distribution in the porous PEEK materials tested by mercury porosimeter are shown in Figure 4. As can be seen in Figure 4, with the increase in the NaCl mass fraction, the pore size of the porous PEEK samples prepared based on the FDM process also showed a trend of increasing. This is due to the increase in the NaCl proportion, resulting in a more obvious agglomeration effect and larger pore size after in situ removal. The smallest pore size is the 2# sample, mostly concentrated in about 0.07 μm, the largest 6# sample pore size reached 7.16 μm, and the pore sizes of all samples were in line with the pore size requirements of the porous bearing cage.

With the increase in the NaCl mass fraction, the porosity of the samples showed a trend of first increasing and then decreasing. The reason for this phenomenon may be that during the preparation of samples 5# and 6#, the large proportion of NaCl led to a large number of agglomerates that affected the uniformity of their own dispersion, which then affected the subsequent porogenesis effect. It can be realized that the porosity can be controlled and the porogenic effect can be improved by arranging the raw material ratio properly.

### 3.3. Oil Content and Oil Retention of the Materials

**Figure 5:**
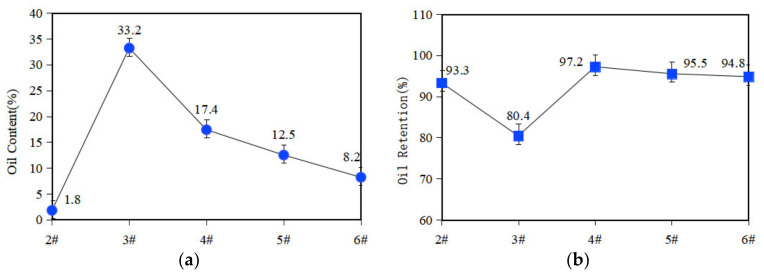
Oil content and oil retention of porous samples. (**a**) Oil content of 2#~6# samples; (**b**) Oil retention of 2#~6# samples.

Oil content and oil retention were important technical indicators for evaluating the performance of oil-containing materials. Five samples of each type of material were tested; the oil content and oil retention test results for each porous PEEK sample are shown in Figure 5.

As can be seen in Figure 5, the oil content of samples 3# and 4# is higher, indicating that the oil content of the porous samples was influenced by their own porosity. The oil retention of the porous PEEK samples prepared based on the FDM process is generally high, and after centrifugation for 2 h, the oil retention of all samples is higher than 80%, except for the 3# sample; the rest of the samples can reach more than 93%, and the porous material shows excellent oil storage performance. The oil retention of sample 3# is higher, but the porosity is limited. Because the interconnected pores are the guarantee to realize the lubricant migration and recovery, the oil retention capacity of sample 3# will be affected. Moreover, the oil retention is an index to consider the oil content of the sample before and after the oil-throwing test. Sample 3# was immersed in the most lubricant in the initial stage, which means the denominator is the largest when calculating the oil retention, and it is difficult to show higher oil retention. However, compared with porous PEEK materials prepared by other processes, the porous material in this paper exhibited more excellent oil retention properties.

### 3.4. Tribological Properties of the Materials

**Figure 6:**
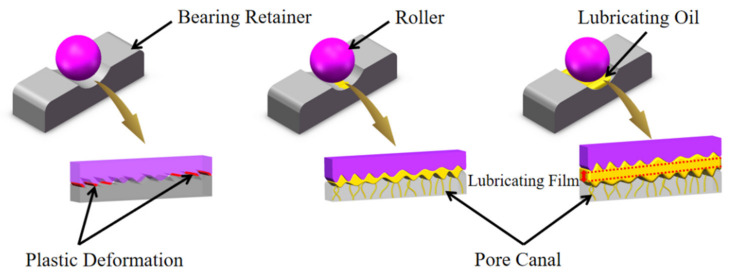
Sliding friction state under different lubrication conditions.

During the rolling bearing operation, sliding friction existed between the cage and the rolling body and inner and outer rings. According to the different lubrication conditions of the friction surface, sliding friction is generally subdivided into dry friction, boundary friction, and fluid friction. These three friction states are shown in Figure 6.

During fluid friction, the surface of the friction pair is completely separated by a layer of pressure fluid, which effectively avoids direct contact with the moving surface and has the best lubrication effect. However, such as in aerospace bearings in ultra-high vacuum, high- and low-temperature alternation, and multiple start–stop and other special conditions, the degree of oil depletion between the friction vice intensified, and was even faced with a dry friction state. Therefore, this paper focused on the dry friction and starved lubrication to research the tribological properties of porous materials.

**Figure 7:**
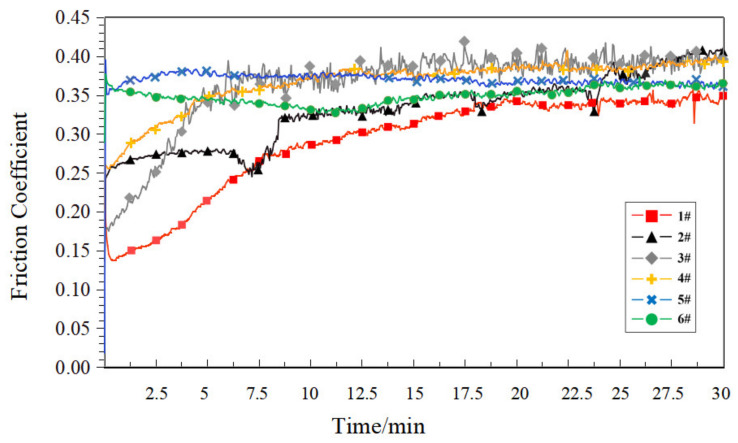
Friction coefficient curve of each sample under the dry friction condition.

Under dry friction conditions, the corresponding friction coefficient curves for each sample are shown in Figure 7.

As can be seen in Figure 7, under dry friction conditions, the wear resistance of the porous materials was closely related to their own porosity. Compact PEEK samples (1#) had relatively smooth surfaces and a minimal coefficient of friction. As the porosity increases, a large number of pores destroyed the smooth surface and the compact structure of the sample, and its surface roughness degree increased. On the other hand, the increase in porosity reduced the mechanical strength of the sample, making the sample more susceptible to wear, creating a more hostile friction environment, and the friction coefficient increased.

**Figure 8:**
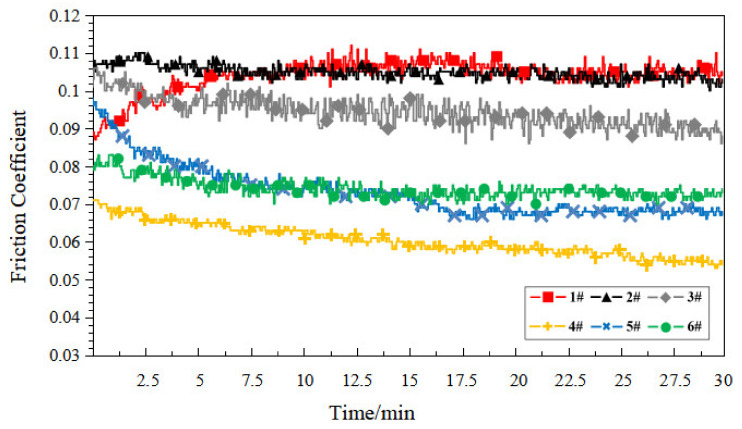
Friction coefficient curve of each sample under the starved lubrication condition.

In order to research the starved lubrication performance of the porous materials, the samples were drained at normal temperature (25 °C) for 48 h. No additional lubricant was added during the test, and the friction coefficient curve of each sample is shown in Figure 8 under the starved lubrication condition.

The internal pores of the porous structure could be used as a lubricant “accumulator” [22]. Under the starved lubrication condition, the lubrication conditions of the friction substrate are affected by the synergistic effect of oil supply and return from the porous surface and pores. As can be seen in Figure 8, the friction coefficient of the compact PEEK sample (1#) was generally higher than that of the porous sample. Additionally, with the increase in porosity, the friction coefficient basically showed a decreasing trend, and the porous sample exhibited prosperous self-lubrication performance. This showed that the higher the porosity of the sample, the stronger its oil supply and return capacity, and the more easily it shows prosperous lubrication. Sample 4# showed the lowest friction coefficient, and the friction coefficient curve was smooth, which was due to its higher oil content and oil retention capacity, which laid the foundation for it to show better self-lubrication performance. It is worth noting that, compared to the compact PEEK sample (1#), the friction coefficient of the porous sample in the starved lubrication condition did not show a significant increase throughout the test, which indicates that the porous PEEK sample prepared based on the FDM process has the potential to provide a stable lubrication environment for the service object to ensure its long-lasting lubrication.

Yang [27] et al. researched the effect of heat treatment conditions on the crystallinity and mechanical properties of PEEK materials. The experimental results showed that the implementation of the heat treatment process can effectively improve the crystallinity of the material surface. In general, the improvement of the surface crystallinity of the polymer materials has a positive significance for improving their surface hardness and wear resistance [30,31]. In order to improve the tribological properties of the cage material, in this paper, the samples were heat treated, and the tribological properties of the materials were studied around the two states of dry friction and starved lubrication.

**Figure 9:**
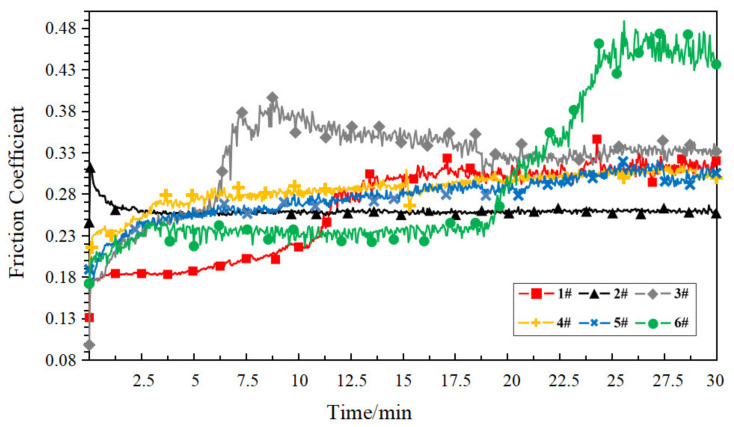
Friction coefficient curve of each heat-treated sample under the dry friction condition.

Figure 9 shows the dry friction coefficient curves of each heat-treated sample. Compared to Figure 7, the friction coefficient distribution range of each sample has been reduced to varying degrees. This is because the heat treatment process leads to an increase in the crystallinity of the PEEK material surface, which was consistent with the conclusion expressed in [31]. It is noteworthy that the friction coefficient curves of the heat-treated samples were not stable compared to the non-heat-treated samples under the dry friction condition, with the friction coefficient curves of samples 1# and 6# showing a large degree of fluctuation after ten minutes of the test. This is because, during the friction test, the abrasive dust formed by wear was retained in the groove, and it has a negative influence on the stability of the subsequent friction coefficient. The heat treatment increases the hardness of the material and forms harder abrasive dust, which could have a more drastic effect on the stability of the friction coefficient.

**Figure 10:**
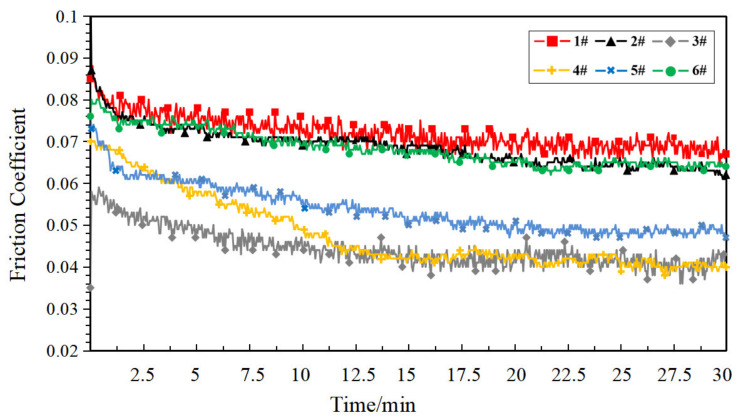
Friction coefficient curve of each heat-treated sample under the starved lubrication condition.

Figure 10 shows the friction coefficient curves for each heat-treated sample under the starved lubrication condition. Compared to the starved lubrication test of the non-heat-treated samples and the dry friction test of heat-treated samples, the friction coefficient distribution of each sample in this test was reduced, and the curve trend was generally smooth. In particular, the porous material (samples 2# to 6#) combined the “double advantage” of the pore structure as the lubricant “accumulator” and the tribological properties bestowed by the heat treatment process to achieve a significant reduction in the friction coefficient.

Taking the friction coefficient arithmetic average value outputted by the equipment as the average friction coefficient, the average friction coefficients corresponding to each type of sample are shown in Table 5.

**Figure 11:**
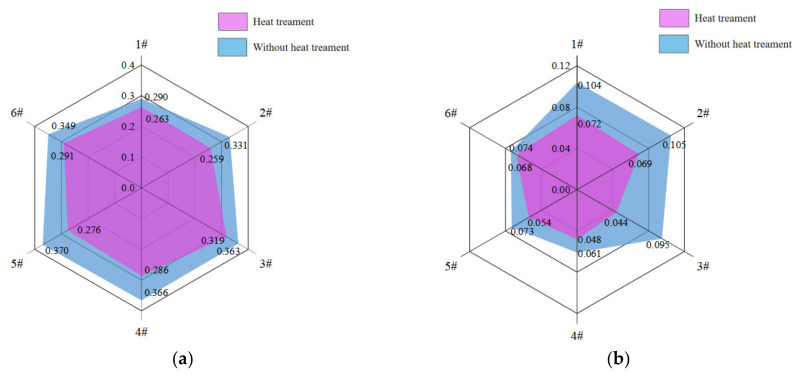
Average friction coefficient of samples 1#~6# under different lubrication conditions. (**a**) Dry friction; (**b**) Starved lubrication.

The following comparative analysis is performed based on four groups of the average friction coefficient. The average friction coefficient for the dry friction and starved lubrication conditions were compared as shown in Figure 11.

Six equally spaced axes were used to represent samples from 1# to 6#, and the samples were divided into two groups according to whether they were heat-treated or not. The points corresponding to the average friction coefficient values of each group were determined on the axes and connected in turn. Obviously, the smaller the hexagon in Figure 11, the smaller the corresponding friction coefficient of the sample. It is clear from Figure 11 that the implementation of the heat treatment process resulted in the better tribological performance of each sample under different lubrication conditions.

**Figure 12:**
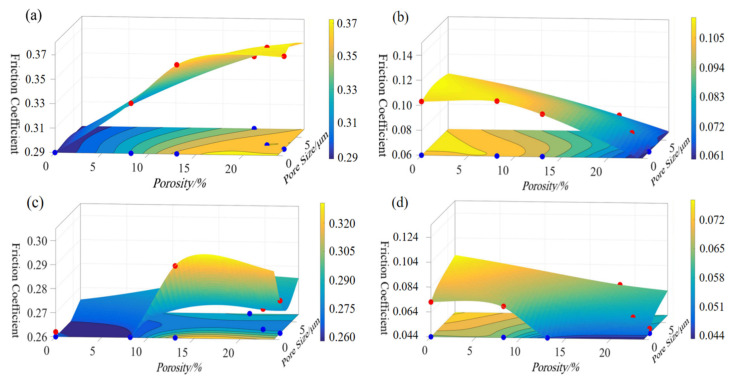
Average friction coefficient of all the non-heat-treated samples and heat-treated samples. (**a**,**b**) Non-heat-treated samples; (**c**,**d**) Heat-treated samples.

Comparing the lubrication conditions of dry friction and starved lubrication, it can be seen from Figure 12 that under the influence of a lubricating medium, the friction coefficient of 2# to 6# decreases more significantly than that of 1#, and this rule was reflected in the friction test of all the samples. For the heat-treated samples, the average friction coefficient with the starved lubrication condition of 1# sample decreased by 64.14% compared to the dry friction condition, while the average coefficient of friction of the samples from 2# to 6# decreased by 76.90% on average, including that porous sample 4# decreased by 83.33%. For the non-heat-treated samples, the average friction coefficient with the starved lubrication condition of sample 1# decreased by 72.62% compared to the dry friction condition, while the average coefficient of friction of the samples from 2# to 6# decreased by 79.97% on average, including that the porous sample 4# decreased by 86.21%. In the dry friction test, the smoothness of the material surface played a dominant role in the friction coefficient, so the friction coefficient of the compact PEEK material was lower, the porous materials were higher, and the friction coefficient was positively correlated with the porosity size. In addition, the dry friction factor of sample 6# (porosity is 18.33%) was significantly lower than that of sample 3# (porosity is 12.92%), no matter whether the heat treatment process had been applied. This phenomenon may be due to the larger surface pore size and lower strength of the pore wall of sample 6#, which makes it easier to form fine abrasive chips to block the pore channel during the dry friction process to form a surface “densification” effect and reduce its own friction coefficient. In the starved lubrication test, the pore canal could release and migrate the lubricant under the action of centrifugal force and other factors, which improved the lubrication between the friction pairs and reduced the friction coefficient. However, compact PEEK materials did not have the above advantage, so sample 1# had a higher friction coefficient. After heat treatment, the crystallinity of the material increases, and the surface properties of the samples improve, so the above rule was reflected to a higher degree in the friction test of heat-treated samples.

**Figure 13:**
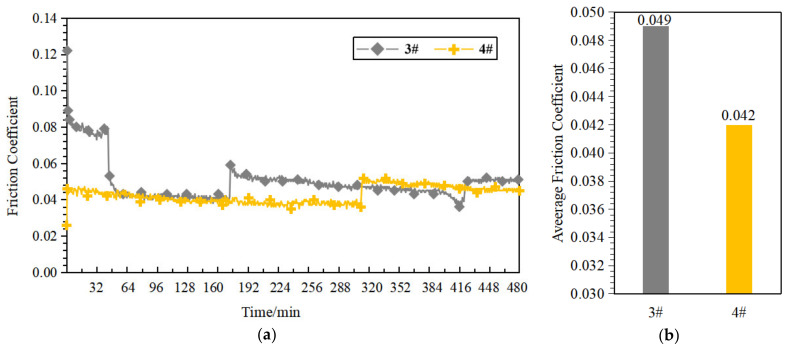
Long-term tribological performance of samples 3# and 4#. (**a**) Friction coefficient curve; (**b**) Average friction coefficient.

The above results show that the application of the heat treatment process can significantly improve the self-lubricating properties of the porous PEEK samples, and the self-lubricating effect is most significant for samples 3# and 4# after heat treatment. To explore the optimal process parameters in this study, we further investigated the long-term tribological performance of the 3# and 4# samples by referring to [32], and the friction test duration was adjusted to 8 h, with the other conditions remaining unchanged. Under dry friction conditions, the friction substrate environment tends to be stable, while under oil-poor lubrication conditions, the long-term friction test is also a test for the oil retention performance of the material. Therefore, we only tested the long-term tribological performance of the target samples under starved lubrication conditions. Figure 13 shows the friction coefficient curves of samples 3# and 4# after heat treatment.

It can be seen in Figure 13 that the friction coefficient of sample 4# was lower and the friction coefficient curve was smoother for the tribological test duration of 8 h, which indicates that sample 4# has better long-term tribological performance. Combining the above results, sample #4 expressed better self-lubricating properties after heat treatment, so the best preparation process parameters in this study were the 60% mass fraction of NaCl, the 40% mass fraction of PEEK, and the imposed heat-treatment process. Compared to [21,29,32], the porous PEEK bearing cage material in this study exhibited better self-lubricating properties and could match the requirements of the cage material.

For the porous materials, the macroscopic tribological properties are subject to the synergistic effects of several factors such as the density, pore size, and porosity of the material [18,33,34,35], and it is difficult to analyze any one factor’s influence independently in the actual analysis process. The limitation of this study is that we only researched the influence of pore size and porosity on the tribological properties but have not explored other factors. In addition, the service life of the bearing cage is determined by the wear rate, which depends on the mechanical properties of the material and the friction characteristics. Therefore, the mechanical properties of the materials prepared in this study need to be further researched.

## 4. Conclusions

(1) Porous materials with different porosities were prepared based on the FDM process with NaCl as the porogenic agent and PEEK as the base material, and the porous materials were heat treated. During the preparation of composite filaments, the filaments with a high percentage of NaCl can be extruded by adjusting the heating temperature and reducing the screw speed; all samples can be prepared by the same printing parameters, and the pore size distribution of the porous samples is below 10 μm, which meets the pore size requirements of the cage materials.
(2) Under the premise of meeting the pore size requirements, the oil content of porous samples prepared based on the FDM process was influenced by their own porosity, and samples with higher porosity also had higher oil contents and exhibited higher oil retention.
(3) The porous cage material prepared by the FDM process exhibited the following tribological properties: under dry friction conditions, the higher the porosity of the porous material, the higher the friction coefficient. The friction coefficient of each sample showed the same pattern after heat treatment, and the friction coefficient of each sample decreased compared to that before heat treatment; under starved lubrication conditions, the friction coefficient of the porous PEEK material decreased significantly compared to that of the compact PEEK material, showing a good self-lubrication effect, and the porous samples reached the best self-lubrication effect after heat treatment. In this study, the optimal process parameters were the 60% mass fraction of NaCl, the 40% mass fraction of PEEK, and the applied heat-treatment process.

## Figures and Tables

**Table 1:** NaCl/PEEK material composition.

Materials	Sample Number					
	1#	2#	3#	4#	5#	6#
PEEK mass fraction/%	100	60	50	40	30	20
NaCl mass fraction/%	0	40	50	60	70	80

**Table 2:** Main process parameters of the wire extrusion process.

Type of Process Parameters	Sample Number					
	1#	2#	3#	4#	5#	6#
Screw speed/r·min^−1^	30	36	43	40	27	18
Zone 1 temperature/°C	327	329	328	326	322	320
Zone 2 temperature/°C	339	343	344	333	328	323
Zone 3 temperature/°C	343	347	348	338	331	325
Zone 4 temperature/°C	345	351	351	338	331	322
Zone 5 temperature/°C	345	348	351	338	333	327
Zone 6 temperature/°C	343	347	348	338	335	328
Zone 7 temperature/°C	340	344	344	331	332	322

**Table 3:** Elongation at break of the corresponding filament for each sample.

Corresponding Samples	Distribution Range/%	Average Value/%
1#	21.54~22.01	21.74
2#	12.31~12.48	12.43
3#	10.30~12.04	11.21
4#	9.85~9.96	9.91
5#	9.69~10.65	9.88
6#	3.21~3.27	3.23

**Table 4:** Percent elemental content of porous samples.

Element	1#	2#	3#	4#	5#	6#
C	75.83	86.97	83.32	83.76	86.84	88.51
O	24.17	12.46	16.67	16.07	12.95	10.83
Na	0.00	0.26	0.00	0.16	0.00	0.00
Cl	0.00	0.31	0.01	0.01	0.22	0.67

**Table 5:** Average friction coefficient of all samples.

Sample Number	Non-Heat-Treated Samples		Heat-Treated Samples	
	Dry Friction	Starved Lubrication	Dry Friction	Starved Lubrication
1#	0.290	0.104	0.263	0.072
2#	0.331	0.105	0.259	0.069
3#	0.363	0.095	0.319	0.044
4#	0.366	0.061	0.286	0.048
5#	0.370	0.073	0.276	0.054
6#	0.349	0.074	0.291	0.068

## Data Availability

The data presented in this study are available on request from the corresponding author.
